# In memoriam: Malcom A.S. Moore (1944–2025)

**DOI:** 10.1038/s41375-025-02806-0

**Published:** 2025-11-06

**Authors:** Karl Welte, Roland Mertelsmann

**Affiliations:** 1 University Children’s Hospital Tuebingen, Tuebingen, Germany; 2 University Clinic Freiburg, Freiburg, Germany


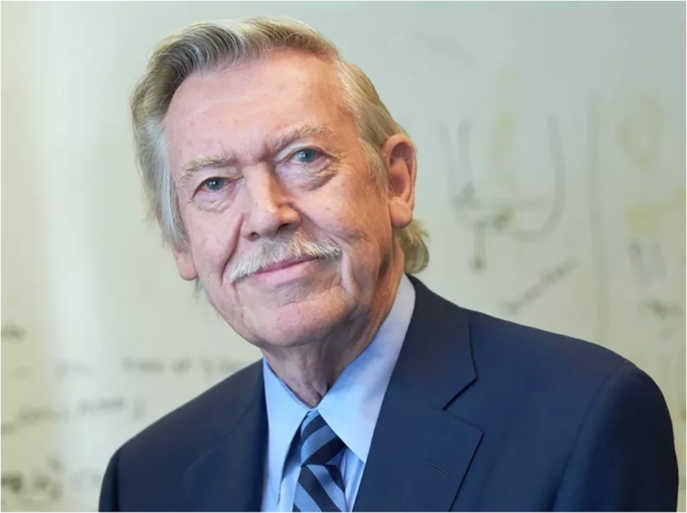
It is with great sorrow that we mark the passing of the brilliant Malcolm A.S. Moore, D.Phil. and M.A., Emeritus Member of the Cell Biology Program in the Memorial Sloan Kettering Cancer Center, New York, on September 23, 2025, in New York City.

Dr. Moore was born in the United Kingdom in 1944 and earned his doctorate from Oxford University in 1967. During his time there, he was appointed the youngest ever Magdalen College, Oxford, Prize Fellow. After graduating, he moved to Melbourne, Australia, where he was a Queen Elizabeth II Visiting Fellow in Donald Metcalf’s laboratory. He then became Head of the Laboratory of Developmental Biology at the Cancer Research Unit of the Walter and Eliza Hall Institute in Melbourne, Australia. In 1974, he joined the Memorial Sloan Kettering Cancer Center in New York as Professor of Biology, Member, and Head of the James Ewing Laboratory of Developmental Hematopoiesis at the Sloan Kettering Institute for Cancer Research. In 1989, he took up the Enid A. Haupt Chair of Cell Biology.

His distinguished career was characterized by pivotal contributions to the fields of hematology and cell biology, including work on growth factors in leukemia cells, on cells of the hematopoietic system, and cells of the immune system, such as interleukin 2, and colony-stimulating factors (CSFs). His most notable achievements were the identification of the responsiveness of leukemic cells to hematopoietic growth factors in the early 1970s [1] and his involvement in the purification and development of G-CSF [2], leading to the drug Filgrastim, in the 1980s [3]. This discovery was named a “Major Cancer Milestone” by the American Society of Clinical Oncology (ASCO). The drug stimulates white blood cell production in people with low counts, often as a side effect of chemotherapy, and has boosted the immune systems of millions of people worldwide.

Dr. Moore received prestigious awards such as the William B. Coley Award for Basic and Tumor Immunology from the Cancer Research Institute, the Cancer Research and Treatment Fund’s Lifetime Achievement Award, the Stratton Award from the American Society for Hematology, the C. Chester Stock Award, Memorial Sloan Kettering Cancer Center, and, as President, the Hope Funds for Cancer Research Clinical Award. He chaired or served on the boards of many well-regarded organizations, including the Cancer Research Institute and Cancer Research &Treatment, and was a member of numerous renowned national and international societies.

From a personal perspective, Malcolm was an exceptional mentor, always supportive with truly brilliant advice, providing intellectual insights into the complexities of cell biology and even human leukemia. Not being a clinician himself, he had the unique and outstanding ability to provide a rational approach to some very complex clinical courses of leukemia, which frequently resulted in clinically relevant insights. He was a wonderful friend, colleague, and mentor to a large community of scientists from around the globe. We extend our heartfelt condolences to Dr. Moore’s family, including his wife, Francine, his son Andy, a fellow cell biologist who is continuing his legacy, as well as his friends and colleagues.
